# Nanomaterial Synthesis in Ionic Liquids and Their Use on the Photocatalytic Degradation of Emerging Pollutants

**DOI:** 10.3390/nano11020411

**Published:** 2021-02-05

**Authors:** Raquel Corchero, Rosario Rodil, Ana Soto, Eva Rodil

**Affiliations:** 1 CRETUS Institute, Department of Chemical Engineering, Universidade de Santiago, E-15782 Santiago de Compostela, Spain; raquel.corchero@rai.usc.es (R.C.); ana.soto@usc.es (A.S.); 2 Department of Analytical Chemistry, Nutrition and Food Science, Universidade de Santiago de Compostela, E-15782 Santiago de Compostela, Spain; rosario.rodil@usc.es

**Keywords:** ionic liquids, nanocatalyst, atenolol, kinetics, degradation pathways

## Abstract

The unique properties of ionic liquids make them suitable candidates to prepare nanoscale materials. A simple method that uses exclusively a corresponding bulk material and an ionic liquid—in this case, [P_6,6,6,14_]Cl—was used to prepare AgCl nanoparticles and AgCl@Fe_3_O_4_ or TiO_2_@Fe_3_O_4_ magnetic nanocomposites. The prepared nanomaterials were characterized by X-ray powder diffraction, scanning electron microscopy, transmission electron microscopy, ultraviolet–visible spectroscopy, and X-ray photoelectron spectroscopy. The photodegradation of atenolol as a model pharmaceutical pollutant in wastewater was investigated under ultraviolet–visible light irradiation using the different synthesized nanocatalysts. In the presence of 0.75 g·L^−1^ AgCl nanoparticles, a practically complete degradation of 10 ppm of atenolol was obtained after 30 min, following pseudo-first-order reaction kinetics. The effect of different variables (concentrations, pH, oxidant agents, etc.) was analyzed. The recyclability of the nanocatalyst was tested and found to be successful. A degradation mechanism was also proposed. In order to improve the recovery stage of the nanocatalyst, the use of magnetic nanocomposites is proposed. Under the same experimental conditions, a slightly lower and slower degradation was achieved with an easier separation. The main conclusions of the paper are the suitability of the use of ionic liquids to prepare different nanocatalysts and the effectiveness of these at degrading an emerging pollutant in wastewater treatment.

## 1. Introduction

Effluents containing emerging pollutants, specifically pharmaceuticals and personal care products (PPCPs) (ibuprofen, atenolol, carbamazepine, etc.), can be detrimental to nature and health [1,2,3]. Different entry paths of these compounds into the environment are known, among which urban wastewater and hospital effluents are considered the most significant [2,4]. Traditional wastewater treatment plants are not designed to remove them [4,5,6,7,8]. This concerns scientific and environmental agencies as these compounds are reaching rivers and canals. Gavrilescu et al. reported the concentration of these compounds in rivers around the world. In particular, European rivers have on average 14–44 ng·L^−1^ of ibuprofen, 314 ng·L^−1^ of atenolol, and 9–157 ng·L^−1^ of carbamazepine, among others [9].

These facts have aroused great interest in investigating wastewater treatment techniques that remove or degrade PPCPs, including filtration [10,11], adsorption [12,13], coagulation/flocculation [14], biological processes [15,16], and degradation by various advanced oxidation processes (AOPs) such as photo-Fenton [17,18], ozonation [19,20], ultrasound treatment [21], electrochemical oxidation [22,23], ultraviolet (UV)/H_2_O_2_ [24,25], etc. Among the AOPs, degradation using ultraviolet–visible (UV–Vis) irradiation and nanomaterials as photocatalytic agents must be highlighted due to the promising results obtained up to now [26,27,28,29,30,31]. Advantages of this method include application at low temperatures and ambient pressures, low environmental impact, easy mineralization of contaminants, and low operating costs [32]. In this research line, Hapeshi et al. [28] studied the degradation by UV irradiation of ofloxacin and atenolol with TiO_2_ nanoparticles as catalysts. Martinez et al. [29] researched degradation of carbamazepine using P-25, anatase, rutile, ZnO, and multi-walled carbon nanotubes–anatase composites as catalysts. The load and type of catalyst and the effect of adding O_2_ or H_2_O_2_ were also analyzed by these authors. Karunakaran et al. [30] removed carboxylic acids from water using UV irradiation with Al_2_O_3_ and SiO_2_ nanoparticles. Ji et al. [31] studied the degradation of atenolol in aqueous TiO_2_ suspensions using a high-pressure mercury lamp as a source of radiation.

The number of possible nanocatalysts for degradation of PPCPs is high, and their synthesis methods varied [33,34]. However, the main drawback in the use of nanomaterials in wastewater treatment is the separation step, owing to the high operation costs. To solve this issue, magnetic nanoparticles or nanocomposites are being proposed [35,36]. Iron oxide and titanium oxide [37,38], ZnO/AgI/Fe_3_O_4_ nanocomposite [39], or FeO and ZnO [40] were easily separated and re-used without losing their photocatalytic activity in different applications.

In recent years, ionic liquids (ILs) have been shown as task-specific solvents of great interest in the preparation of different nano-scale materials [41,42,43,44,45,46]. AgCl [47], Ag [48], TiO_2_ [49], and CeO_2_ [50], among other nanoparticles, have been successfully synthesized using these neoteric solvents. Even though the preparation methods are different, all of them share the advantages of ILs, mainly their green character (atmospheric contamination is avoided with the use of these salts) and their tunable character. Chen et al. [51] used a solvothermal process to synthesize the photocatalyst bismuth phosphate (BiPO_4_) with different morphologies in the presence of the ionic liquid [Omim]H_2_PO_4_. Its photocatalytic performance was tested under ultraviolet irradiation for the elimination of ciprofloxacin as a target contaminant. Xia et al. [52], using the same method, synthesized the g-C_3_N_4_/BiPO_4_ hybrid material and tested it as a photocatalyst for the removal of methylene blue dye and the antibiotic ciprofloxacin. The photocatalytic degradation of rhodamine B, tetracycline hydrochloride, ciprofloxacin, and bisphenol A was carried out using carbon quantum dot-modified bismuth oxychloride/bismuth oxybromide nanosheet by Hu et al. [53]. To that end, an in situ ionic liquid-induced strategy was used with [C_16_mim]Cl. Using the same IL, Yin et al. [54] synthesized novel carbon quantum dot-modified PbBiO_2_Cl for degradation of tetracycline hydrochloride, ciprofloxacin, and bisphenol A. BiOBr microspheres were synthesized [55] in the presence of three different reactive ILs, namely 1-butyl-3-vinylimidazolium bromide, poly(1-butyl-3-vinylimidazolium) bromide, and poly(1-butyl-3-vinylimidazolium bromide acrylamide. Photocatalytic activity of the microspheres was tested with rodamine B and tetracycline. All these nanocatalysts [51,52,53,54,55] have shown good results in the degradation of pharmaceuticals; however, their synthesis is complicated and requires different solvents besides the IL.

In this work, AgCl nanoparticles and magnetic nanocomposites (AgCl@Fe_3_O_4_ and TiO_2_@Fe_3_O_4_) are prepared using the IL trihexyl(tetradecyl)phosphonium chloride ([P_6,6,6,14_]Cl) and the corresponding bulk materials. The selected method of preparation [47,56] is quick and easy. The synthesized nanomaterials are characterized by X-ray powder diffraction (XRD), transmission electron microscopy (TEM), scanning electron microscopy (SEM), ultraviolet–visible spectroscopy (UV–Vis), and X-ray photoelectron spectroscopy (XPS). Atenolol (ATL) is used as a pharmaceutical pollutant model, and AgCl nanoparticles are used as catalysts in its photodegradation with UV light. Several parameters are evaluated: nanocatalyst loading, atenolol concentration, addition of oxidant agents, and pH. The kinetics of the degradation process are determined by measuring the variation of the ATL concentration with time using high-performance liquid chromatography (HPLC), and the degradation products are identified. Furthermore, recyclability of the nanocatalyst is shown. Finally, the synthesized magnetic nanocomposites, prepared with the aim of facilitating catalyst recovery, are also tested under the same conditions as the individual nanoparticles.

## 2. Materials and Methods

### 2.1. Materials

**Figure 1:**
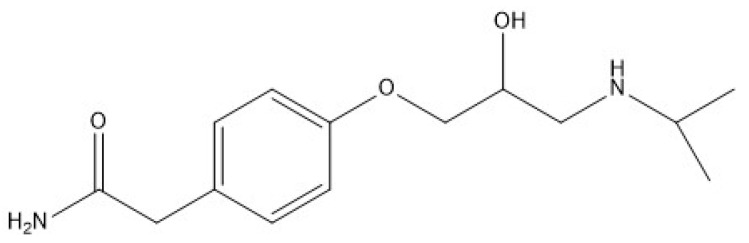
Structure of atenolol (ATL).

**Figure 2:**
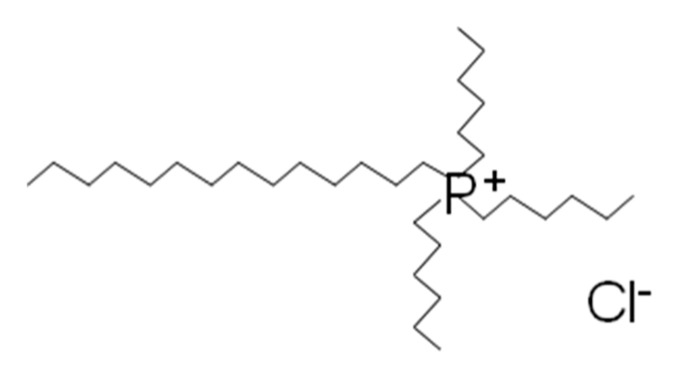
Structure of [P_6,6,6,14_]Cl.

ATL (>98%), whose structure is shown in Figure 1, magnetite (97%, nanopowder 50–100 nm), titanium (IV) oxide (>99.5% P25 degussa, nanopowder), silver chloride (99%), toluene (≥99.5%), sulfuric acid (72%), acetic acid (99%), hydrogen peroxide (30% in H_2_O), acetone (≥99.5%), and ammonia (25%) were obtained from Merck / Sigma-Aldrich (Germany). Ethanol (99.8%) and sodium hydroxide were purchased from Panreac, and methanol (99.9%, HPLC) was supplied by Scharlau (Barcelona, España). [P_6,6,6,14_]Cl, was obtained from CYTEC industries (Woodland Park, New Jersey, United States) under the trade name CYPHOS IL 101 (97.7%); Figure 2. The IL was dried at 70 °C under high vacuum (absolute pressure < 1 Pa) for 24 h and then stored under inert atmosphere. The water content (<2000 ppm) was measured by titration using a Metrohm 737 Karl Fischer coulometer. IL final purity was checked by ^1^H and ^13^C NMR analyses (Figure S1).

### 2.2. Preparation of AgCl Nanoparticles

AgCl nanoparticles were synthesized using a previously published method [56]. In a round-bottom flask, a certain amount of bulk AgCl was mixed with [P_6,6,6,14_]Cl to obtain a concentration of 10% *w*/*w*. The mixture was stirred vigorously at 120 °C for 4 h. Then, ethanol was used to precipitate the nanoparticles. They were washed three times with acetone to remove any possible trace of IL. Finally, the nanoparticles were dried at 80 °C for 12 h in the dark.

### 2.3. Preparation of AgCl@Fe_3_O_4_ and TiO_2_@Fe_3_O_4_ Magnetic Nanocomposites

The procedure above mentioned [56] was used, for the first time, to synthesize nanocomposites (AgCl@Fe_3_O_4_ and TiO_2_@Fe_3_O_4_). Commercial magnetic nanoparticles of Fe_3_O_4_ were dissolved in pure [P_6,6,6,14_]Cl to obtain a 5% *w*/*w* concentration. When a homogeneous solution was obtained, the chosen photocatalytic nanomaterial (synthesized AgCl or commercial TiO_2_ nanoparticles) was added at a 5% *w*/*w* concentration. The mixture was stirred at 120 °C for 4 h. Precipitation and washing procedures were performed as described above.

### 2.4. Characterization of the Nanomaterials

The prepared nanomaterials were structurally characterized by XRD. The diffraction patterns were obtained using an X-ray Philips powder diffractometer (PW 1710) with a Cu-kα X-ray source (λ = 1.54 Å). SEM and TEM were used to determine the shape and size of the nanomaterials. One drop of dispersed nanoparticles in toluene was deposited on a 400 mesh carbon formvar grid and allowed to evaporate at room temperature. SEM images were obtained with a field emission scanning electron microscope Zeiss Ultra Plus FESEM, with energy-dispersive X-ray micro-analysis (EDS). TEM images were obtained using a Philips CM-12 microscope (FEI Company, Eidhoven, The Netherlands) with a MegaView docu-II camera and IMAX image analysis Software SIS NT. The UV–Vis absorption spectra of nanoparticles dispersed in toluene were obtained with an Agilent 8543 absorption spectrophotometer. Finally, to confirm the surface composition and chemical states of the nanocatalysts before and after usage, a Thermo Scientific K-Alpha ESCA instrument equipped with an aluminum Kα monochromatized radiation at 1486.6 eV X-ray source (XPS) was used.

### 2.5. Experimental Set-Up for Photocatalytic Degradation of ATL

**Figure 3:**
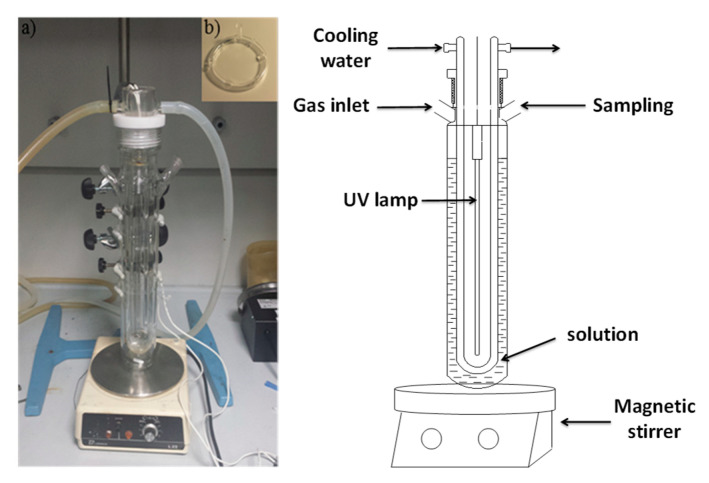
Glass photoreactor picture and scheme.

The photocatalytic performance of the obtained nanomaterials was evaluated by degrading 10 ppm ATL stock solutions. The experiments were carried out in a 250-mL designed glass photoreactor (Figure 3) equipped with a low-pressure mercury vapor lamp (UV-C, λ = 280–100 nm). Furthermore, the reactor was equipped with a double stirring system consisting of a magnetic stirrer and a gas diffuser. The reaction camera including the irradiation source was surrounded by a quartz cooling jacket to control the temperature.

The nanomaterial, dispersed in water solution, was added to the ATL stock solution to obtain a concentration of 0.75 g·L^−1^ (except when this effect was evaluated where the corresponding concentrations were prepared). Before starting irradiation, the suspension was magnetically and bubble-stirred for 30 min in the dark to achieve adsorption–desorption equilibrium. Then, the UV light was switched on and samples were taken at different time intervals during the reaction, centrifuged (14,500 rpm during 10 min), and immediately analyzed. In the experiments carried out, helium was bubbled in to maintain an inert atmosphere free of oxidizing agents (except when the effect of oxidizing agents was evaluated). All tests were accomplished at least twice, guaranteeing their repeatability.

### 2.6. Analytical Method

The ATL concentration was measured by high-performance liquid chromatography (HPLC) using an Agilent 1100 chromatograph equipped with a diode array detector (λ = 224 nm), vacuum degasser unit, quaternary pump, and thermostated autosampler. Separation was performed by a ZORBAZ SB-C18 column (4.6 × 150 mm; 80 Å pore size) at 30 °C. The isocratic elution was 20/80 (*v*/*v*) methanol/ammonium acetate 10 mM (aq.). Flow rate was set as 0.5 mL/min and the injection volume was 20 µL.

ATL concentration was determined using an area–concentration calibration. Subsequently, the concentration of the samples was measured, and the degradation percentage was calculated according to Equation (1):

(1) $$\%degradation=\left(1-\frac{C}{C_{0}}\right)\cdot 100$$

where *C*_0_ and *C* are the initial and the sample concentration of ATL, respectively.

Furthermore, the degradation products were identified by liquid chromatography–quadrupole-time-of-flight (LC-Q-TOF). To that end, an Agilent 1200 Series HLPC system consisting of a membrane degasser, a binary high-pressure gradient pump, an autosampler, and a thermostated LC column compartment was used. This system was interfaced to a Q-TOF mass spectrometry (Q-TOF-MS) instrument (Agilent 6520 Series) equipped with a dual electrospray ion source. Separation was carried out on a 100 × 2 mm (particle size: 3 μm) Synergi Fusion RP (Phenomenex, Torrance, CA, USA) at a flow rate of 0.2 mL min^−1^ and temperature of 35 °C. Mobile phase consisted of Milli-Q water (A) and methanol (B), both containing 5 mM of ammonium acetate. The gradient was as follows: 0 min, 5% B; 10–12 min, 100% B; 12.10−25 min, 5% B. For the Q-TOF-MS, nitrogen (99.9%), used for nebulizing and drying gas, was provided by a nitrogen generator (Erre Due Srl, Livorno, Italy). Nitrogen (99.9995%) used for collision-induced dissociation was supplied by Praxair Spain (A Coruña, Spain). The electrospray ion source was operated in positive (no transformations products were detected in negative) mode with the following parameters applied: gas temperature: 350 °C; drying gas: 7 L min^−1^; nebulizer: 42 psig; capillary: 4000 V; fragmentor: 120 V; skimmer voltage: 65 V; octopole radio frequency peak: 750 V. The instrument was operated in the 2 GHz (extended-dynamic range) mode, which provides a Full Width at Half Maximum (FWHM) resolution of ca. 4500 at m/z 121 and ca. 11,000 at 922 m/z. A reference solution was also continuously infused using a second nebulizer of the dual electrospray ion source (5 psig) to recalibrate the Q-TOF using two masses (m/z 121.0509 and 922.0098) and maintain mass accuracy. Instrument control, data acquisition, and evaluation were performed with the MassHunter software (Agilent Technologies). Finally, MS/MS analyses were performed using different collision energies (10, 20, and 40 V) and interpreted in order to tentatively elucidate the structure of the degradation products.

## 3. Results and Discussion

### 3.1. Characterization of AgCl Nanoparticles

**Figure 4:**
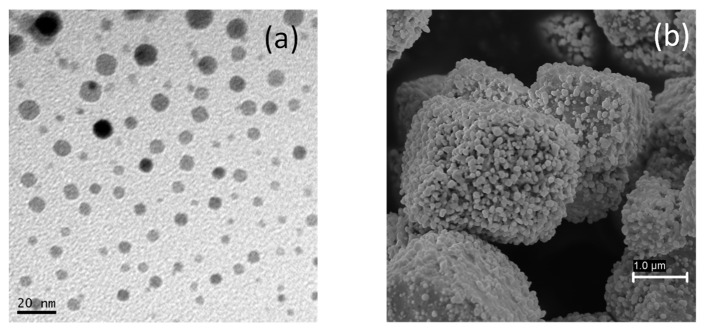
AgCl nanoparticles characterization: (**a**) TEM image, (**b**) SEM image.

The morphology of the AgCl nanoparticles was characterized by SEM and TEM (Figure 4a,b). As shown in Figure 4a, the dispersed AgCl nanoparticles have a regular and spherical shape with homogeneous distribution (5–20nm). Figure 4b shows the SEM image of the precipitated nanoparticles; the solid was formed of large cubic agglomerates, behavior which has previously been reported by other authors [47,57,58]. The UV–Vis absorption spectrum of the dispersed nanoparticles (Figure S2) indicates the presence of silver chloride, with a characteristic absorption peak below 300 nm [59]. Moreover, there are no absorption peaks in the visible light region, which indicates that the photosensitive silver chloride has not been converted into silver (its absorption peak should appear at 400 nm [60]). AgCl nanoparticles were structurally characterized by XRD. The position and relative intensities of the peaks observed in Figure S3 indicate the presence of chlorargyrite only, the cubic structure of silver chloride, with the peaks matching the standard JCPD (Joint Committee on Powder Diffraction Standards) card number 31-1238 [61,62]. The bonding configuration and element analysis were determined by XPS, which allowed confirmation of the surface composition and chemical states of the nanoparticles. Figure S4a shows two band peaks corresponding to Ag 3d3/2 and Ag 3d5/2 at 371.38 and 365.38 eV, respectively. Figure S4b shows the XPS Cl spectrum with two peaks at 197.48 and 195.88 eV, belonging to Cl 2p1/2 and Cl 2p3/2, respectively. These binding energy values and the obtained Ag/Cl ratio of 0.99 indicate that the nanocatalyst is AgCl [63]. 

### 3.2. Characterization of TiO_2_@Fe_3_O_4_ and AgCl@Fe_3_O_4_ Nanocomposites

**Figure 5:**
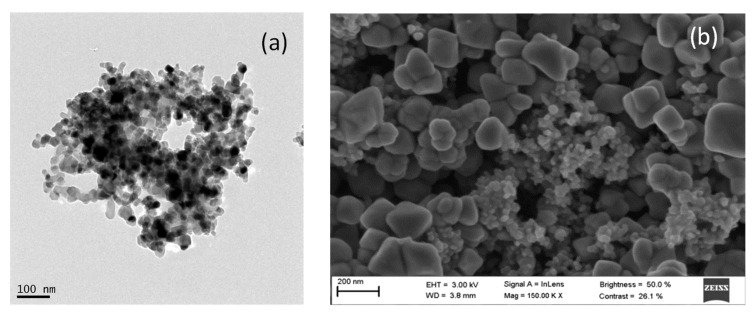
TiO_2_@Fe_3_O_4_ nanocomposite: (**a**) TEM image, (**b**) SEM image.

Figure 5a shows a TEM image for TiO_2_@Fe_3_O_4_. The nanocomposite shows a regular, almost spherical morphology with a size distribution between 20 and 50 nm. Figure 5b and Figure S5 show SEM and EDS spectra, respectively. In Figure S5, besides the peaks corresponding to Fe, O and Ti from the nanocomposite, Cu peaks from the copper grid are also observed. The XRD patterns of the prepared TiO_2_@Fe_3_O_4_ nanocomposite are shown in Figure S6. The diffraction peaks that appear for Fe_3_O_4_ correspond to the standard JCPD card number 39-1346. In the case of TiO_2._ (P25-Degussa, 20% rutile and 80% anatase), the peaks match a standard for rutile, JCPD card number 21-1273, and for anatase, JCPD card number 21-1272. Finally, XPS was used to confirm the surface composition (Ti and Fe) and the chemical states of the nanocomposite. As can be seen, the binding energies in Figure S7a,b can be assigned to Fe_3_O_4_ and TiO_2_. The characteristic peaks of Fe 2p3/2, Fe 2p1/2, Ti 2p1/2, and Ti2p3/2 are at 724, 710, 464.59, and 458.92eV, respectively [38,64]. 

**Figure 6:**
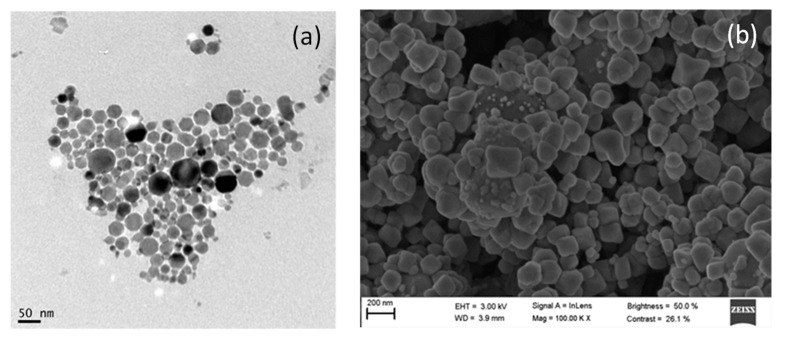
AgCl@Fe_3_O_4_ nanocomposite: (**a**) TEM image, (**b**) SEM image.

Figure 6a shows a TEM image of the AgCl@Fe_3_O_4_ nanocomposite. The formation of mainly spherical aggregates with a size distribution between 10 and 40 nm is observed. Figure 6b and Figure S8 show an SEM image and EDS spectrum. The peaks shown in Figure S8 are those corresponding to Fe, O, Cl and Ag from the nanocomposite, and the Cu peak comes from the copper grid. The XRD patterns of the prepared AgCl@Fe_3_O_4_ nanocomposite are shown in Figure S9. The diffraction peaks for Fe_3_O_4_ correspond to the standard JCPD card number 39-1346, and for AgCl, to the standard JCPD card number 31-1238 (chlorargyrite) [38,61,62,63,64]. Furthermore, XPS surface analysis was also used to confirm the surface composition and chemical states of the nanocomposite. Figure S10a shows the spectrum with binding energies at the characteristic peaks of Ag 3d3/2 and Ag 3d5/2 at 373.91 and 367.92 eV, respectively. Figure S10b shows the peaks of Fe 2p3/2 and Fe 2p1/2 at 724 and 710 eV, respectively [38,63,64]. 

### 3.3. Photocatalytic Degradation of ATL with AgCl Nanoparticles

#### 3.3.1. Degradation Tests

**Figure 7:**
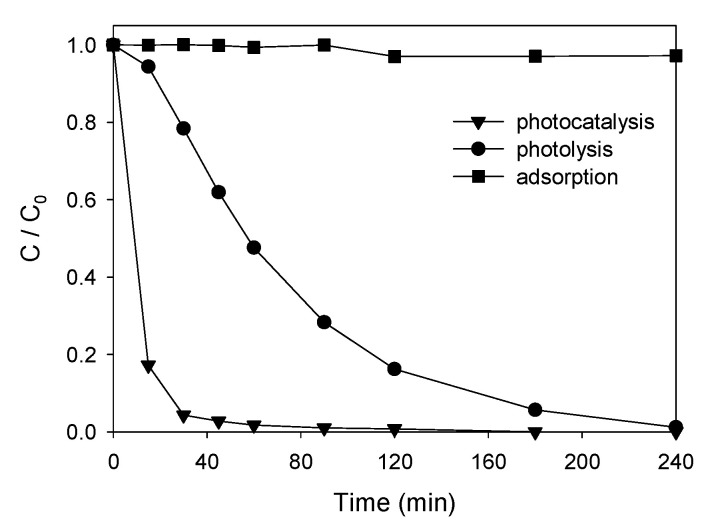
Comparison of ATL degradation methods.

The degradation of an aqueous solution with 10 ppm of ATL was carried out firstly using only UV irradiation, secondly with a nanoparticle concentration of 0.75 g·L^−1^ in the dark, and finally using the same concentration of AgCl nanoparticles under UV irradiation. Concentrations were determined by HPLC. Figure 7 shows the variation of ATL concentration with time for all the cases studied. As shown, in the case of the photolysis process (without catalyst), the degradation was very slow, only 5% and 70% were achieved in 15 and 90 min, respectively. More than 240 min were required to achieve a practically complete degradation. In the case of the study in the dark, a negligible decrease in the concentration, mainly due to adsorption of ATL, was observed. The photocatalytic degradation of ATL with AgCl nanoparticles allowed a total and quick degradation (82% in 15 min, 98% in 45 min). Therefore, the presence of nanoparticles significantly accelerates ATL removal efficiency, decreasing reaction times, energy consumption, and process costs.

**Figure 8:**
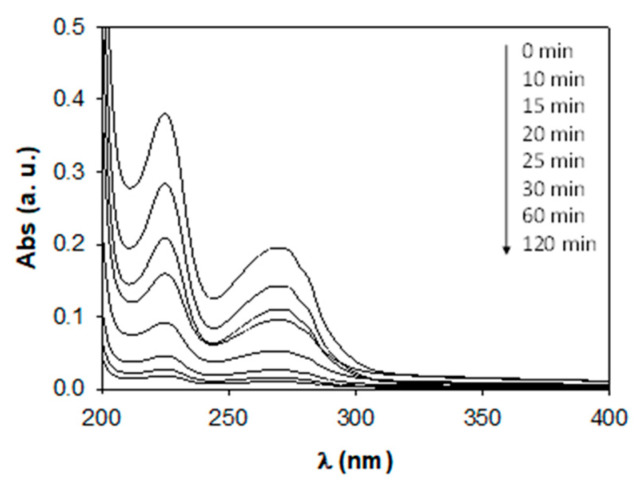
Variation of the UV–Vis absorption spectrum during the degradation of 10 ppm of ATL with 0.75 g·L^−1^ of AgCl.

The degradation of the aqueous solution with 10 ppm of ATL and 0.75 g·L^−1^ of AgCl can be easily observed in the progressive evolution of UV–visible absorption spectra (Figure 8). The remarkable decrease in the absorbance peak at 224 nm over time indicates a very significative degradation of ATL, thus confirming the catalyst’s excellent photocatalytic activity under UV light irradiation.

**Figure 9:**
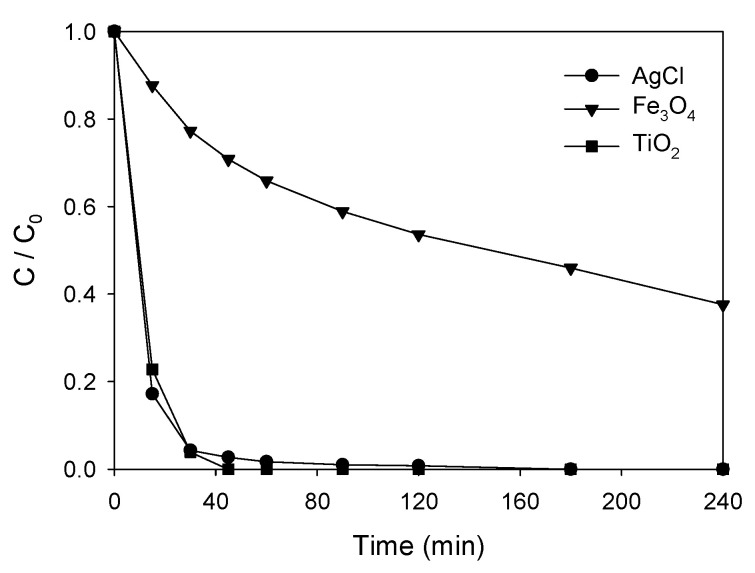
Comparison of nanocatalysts (0.75 g·L^−1^) in the photocatalytic degradation of ATL (10 ppm) in aqueous solutions.

With the aim of comparing the proposed nanocatalyst with other commonly used ones, new tests were carried out using TiO_2_ (P25-degussa) and Fe_2_O_3_ nanoparticles. The results obtained can be seen in Figure 9. A total degradation of ATL was achieved after 45 min in the case of TiO_2_, a slightly higher value than in the case of AgCl that, at the same time, achieved a degradation of about 98%. However, as previously reported [65,66], suspensions of TiO_2_ nanoparticles in water form a highly stable hydrocolloid that makes separation of the nanoparticles from water difficult. Therefore, the recovery and reuse of this nanocatalyst become more complicated and the process becomes less efficient. In the case of Fe_2_O_3_, with a clearly lower reduction in the ATL concentration, the process cannot be considered competitive.

#### 3.3.2. Influence of Operational Parameters

In order to analyze the effect of different variables on the performance of the degradation of ATL, some tests were conducted as reflected in the following subsections.

##### AgCl Concentration

**Figure 10:**
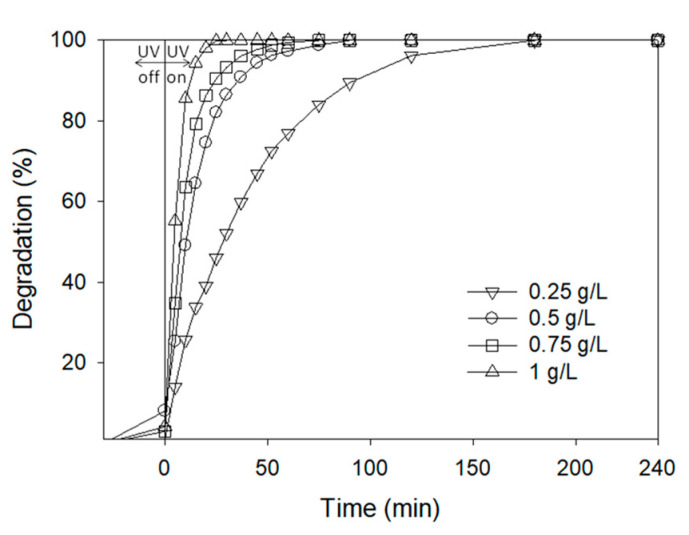
ATL degradation (%) using different concentrations of nanocatalyst. Initial ATL concentration: 10 ppm.

Aqueous solutions of ATL (10 ppm) were submitted to photocatalytic degradation using AgCl nanoparticle concentrations ranging from 0.25 to 1 g L^−1^. Figure 10 clearly shows that at a given time, an increase in nanoparticle concentration causes an increase in the degradation percentage. Total degradation of ATL was achieved in all the experiments. However, the increase in nanocatalyst concentration from 0.25 to 1 g·L^−1^ reduced the required time for complete degradation from 180 to 25 min. As shown in Figure 10, the adsorption before light irradiation has a very small influence on the degradation process.

##### ATL Initial Concentration

**Figure 11:**
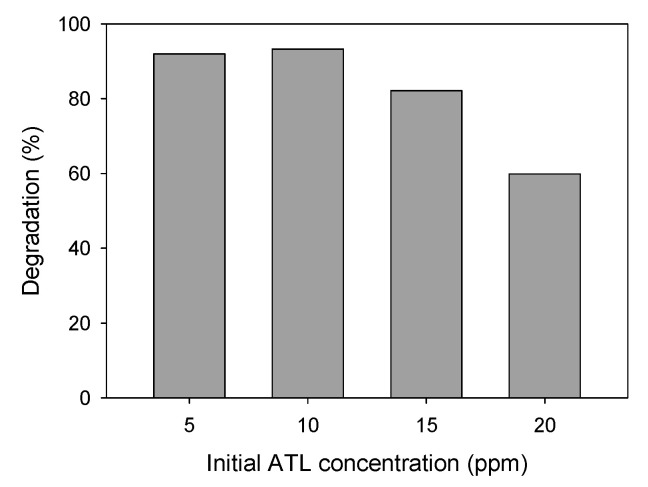
ATL degradation (%) using different concentrations of contaminant. AgCl nanoparticles concentration: 10 ppm.

Aqueous solutions of different concentrations of ATL (ranging from 5 to 20 ppm) were submitted to photocatalytic degradation using 0.75 g·L^−1^ of AgCl as a nanocatalyst. Figure 11 shows the percentage of degradation achieved after 30 min under UV light irradiation (a preliminary period of 30 min in the darkness was always maintained). As shown, degradations of 92%, 93%, 82%, and 60% were obtained for solutions with 5, 10, 15, and 20 ppm of initial ATL concentration, respectively. The effect of this parameter on degradation is not significant up to 10 ppm, but higher concentrations for the same nanocatalytic load and the same exposure time to UV light irradiation are associated with a lower degradation percentage.

##### pH Solution

**Figure 12:**
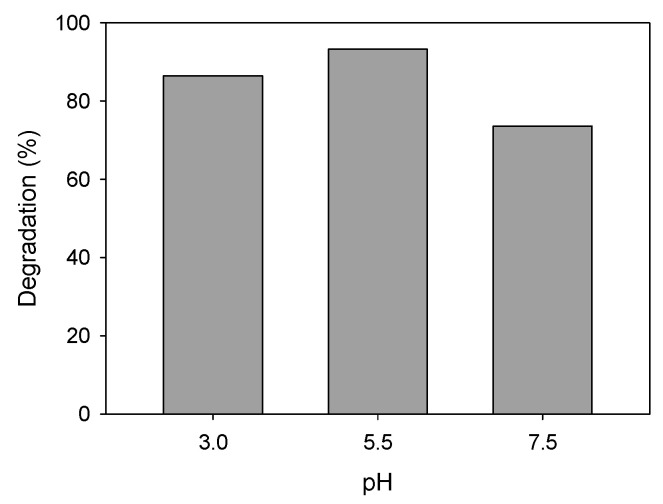
ATL degradation (%) at different pH aqueous solutions. Initial ATL concentration: 10 ppm; AgCl nanoparticles concentration: 0.75 g·L^−1^.

To simulate different types of wastewaters, the pH of the aqueous solutions containing ATL was varied using the required amount of sulfuric acid or sodium hydroxide. The natural pH of 10 ppm ATL solution was 5.5 and it was varied to obtain values of 3 and 7.5. Figure 12 shows the results of degradation (30 min) using 0.75 g·L^−1^ of AgCl as a nanocatalyst. The degradation achieved in a solution at acidic pH was slightly lower than in the case of natural pH (86% and 93%, respectively). With the increase in pH to 7.5 to obtain a slightly basic medium, the degradation decreased to 73%. Similar behavior was previously reported by Bhatia et al. [67].

##### Addition of Oxidizing Agents

**Figure 13:**
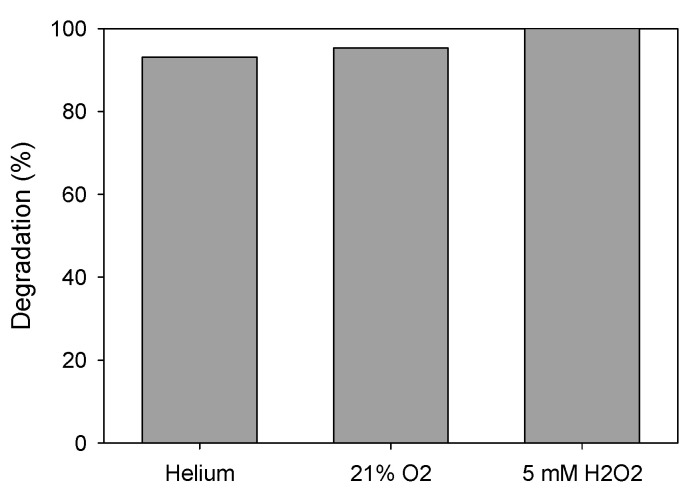
ATL degradation (%) with and without oxidizing agents. Initial ATL concentration: 10 ppm; AgCl concentration: 10 ppm.

To evaluate the effect of the addition of oxidizing agents on the photocatalytic process, besides the standard experiment where helium was bubbled to avoid the presence of oxidizing experiments, two new tests were developed where hydrogen peroxide was added or air (oxygen source) was bubbled. In all the cases, the initial concentration of ATL was 10 ppm and nanoparticle concentration was 0.75 g·L^−1^. When air was bubbled, the degradation at 30 min slightly increased from 93% to 95.5% (Figure 13). When hydrogen peroxide (H_2_O_2_) was added (5 mM), total degradation was achieved in the same period of time. A similar effect was previously found by Li et al. [68].

#### 3.3.3. Durability of the Nanophotocatalyst

**Figure 14:**
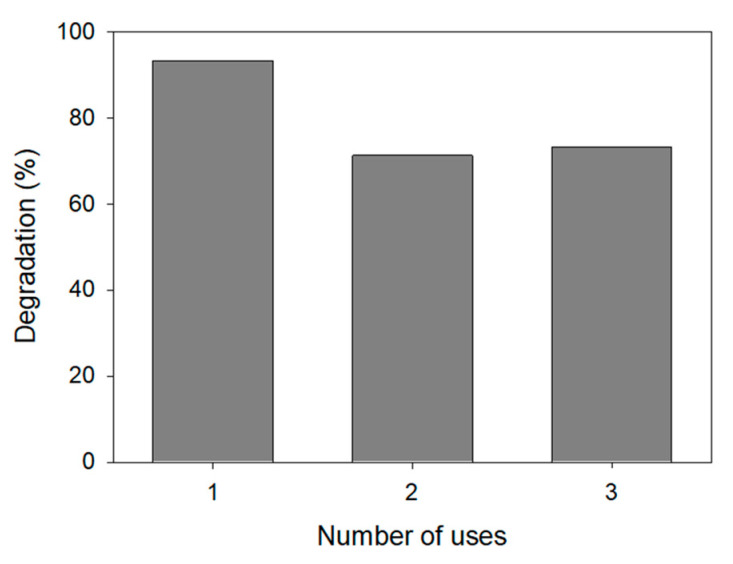
Degradation as a function of the number of uses of the nanocatalyst.

To achieve an economical process, it is important to evaluate the possibility of reusing the catalyst. Three cycles of degradation were carried out to analyze catalyst durability and efficiency. After each run of degradation (10 ppm ATL, 0.75 g·L^−1^ AgCl), the nanoparticles were separated from the aqueous solution by centrifugation, washed with water, and dried at 100 °C for 12 h. The nanocatalyst was then used in a subsequent degradation process under identical conditions. As can be seen in Figure 14, after the first degradation experiment, in which 93% degradation was achieved, the efficiency of the process decreased to 75%. No further decrease was detected. Part of the reduction in the efficiency is likely due to the loss of nanoparticles in the separation process.

#### 3.3.4. Kinetics

**Figure 15:**
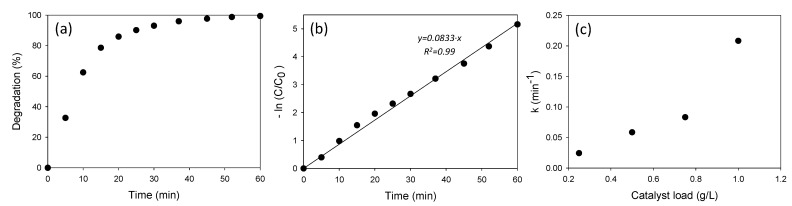
Kinetic studies of photocatalytic degradation of ATL: (**a**) Degradation with time (10 ppm ATL and 0.75 g·L^−1^ of AgCl). (**b**) Fit to a pseudo-first-order reaction. (**c**) Pseudo-first-order coefficients as a function of nanocatalyst load.

The kinetics of the degradation process were studied through the evolution of the ATL concentration with time. The study was carried out (10 ppm ATL, 0.75 g·L^−1^ AgCl) by taking samples of the liquid phase at different times and analyzing them by HPLC. The results are shown in Figure 15a. Obtained data were adjusted to a pseudo-first-order reaction,

(2) $$-ln\left(\frac{C}{C_{0}}\right)=k_{app}\cdot t$$

where *k_app_* is the pseudo-first-order rate coefficient (min^−1^), *t* is the degradation time, and *C* and *C*_0_ are the ATL concentrations at time *t* and at the beginning of the process, respectively. Figure 15b shows −ln(*C/C*_0_) as a function of time. A linear fit, R^2^ > 0.99, led to a pseudo-first-order kinetic coefficient of 0.0833 min^−1^. Kinetic tests were also carried out with the same ATL concentration but different concentrations of nanoparticles (0.25, 0.5, and 1 g·L^−1^). In all cases, the linear fits were satisfactory and pseudo-first-order kinetic coefficients were calculated. The results are shown in Figure 15c. As expected, the coefficient increases with the increase in the nanocatalyst load.

#### 3.3.5. Degradation Products

Degradation products were identified as a means to evaluate possible pathways of AgCl-mediated ATL transformation. The HPLC chromatogram of the irradiated sample revealed some new peaks compared to the non-irradiated sample, which was indicative of the formation of intermediates. In order to determine as many intermediates as possible, aliquots at different irradiation times from the degradation process were analyzed by LC-Q-TOF-MS and MS/MS as described before [69,70]. Briefly, a list of possible entities for each chromatogram was generated using the algorithm “Find by molecular feature”. Then, a comparison between the control group (aliquots at time 0) and aliquots at different times was performed using the MassProfiler Professional software. Next, empirical formulae were generated for the potential degradation products with cut-off values of mass error <5 ppm and score >80 (100 being a perfect match of accurate mass and isotopic distribution). A list of the detected transformation products and identification parameters is shown in Table 1. As shown, the empirical formula can be applied with a high degree of certainty, with score values higher than 85% and mass errors lower than 3.5 ppm.

**Figure 16:**
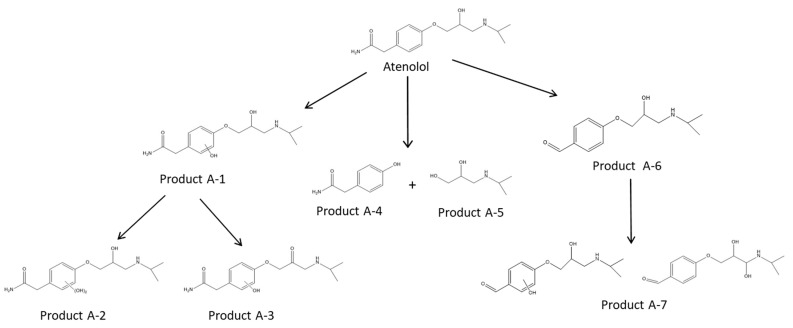
Proposed mechanism for the degradation of ATL.

The proposed structures are based on the interpretation of the MS/MS spectra and the literature (Figure 16). All the identified products have been previously reported in the literature [31,71,72,73]. Products A-1 and A-2 consist of the addition of one or two hydroxyl groups to the aromatic ring. After 40 min, these products were degraded, and at longer exposure times, product A-3 was produced by oxidation of the hydroxyl group of the aliphatic chain to the ketone. Two isomers were observed for these products. Products A-4 and A-5 were formed through hydroxylation followed by the cleavage of the bond between the oxygen and the aromatic carbon. The product A-5 was stable at long degradation times, while product A-4 disappeared. The benzaldehyde derivative product A-6 was produced after the loss of the amide group, hydroxylation, and oxidation. Further hydroxylation of this product resulted in the formation of product A-7. Three isomers of A-7 were observed. Thus, at long reaction times (after 180 min), the most persistent degradation products were A-5 and A-3.

### 3.4. Photocatalytic Degradation of ATL with AgCl@Fe_3_O_4_ or TiO_2_@Fe_3_O_4_ Nanocomposites

**Figure 17:**
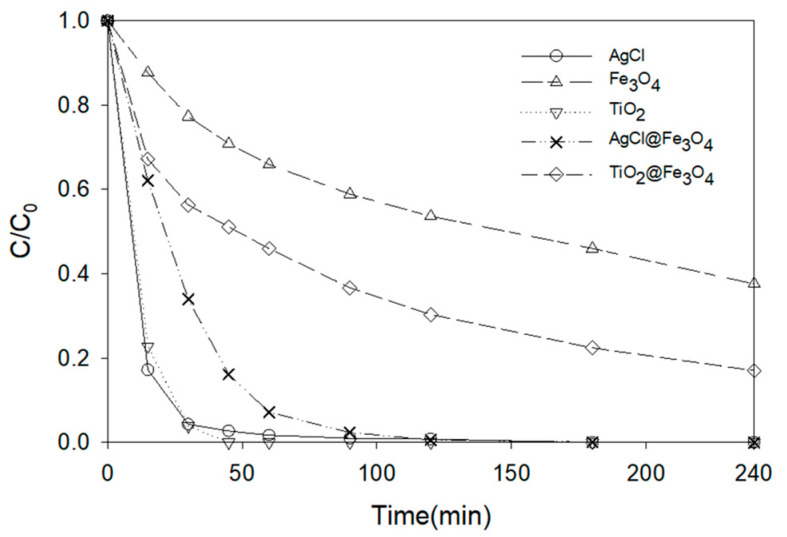
Degradation performances (*C/C*_0_) as a function of time using different nanocatalysts (the lines are only to facilitate the visualization of the results).

**Figure 18:**
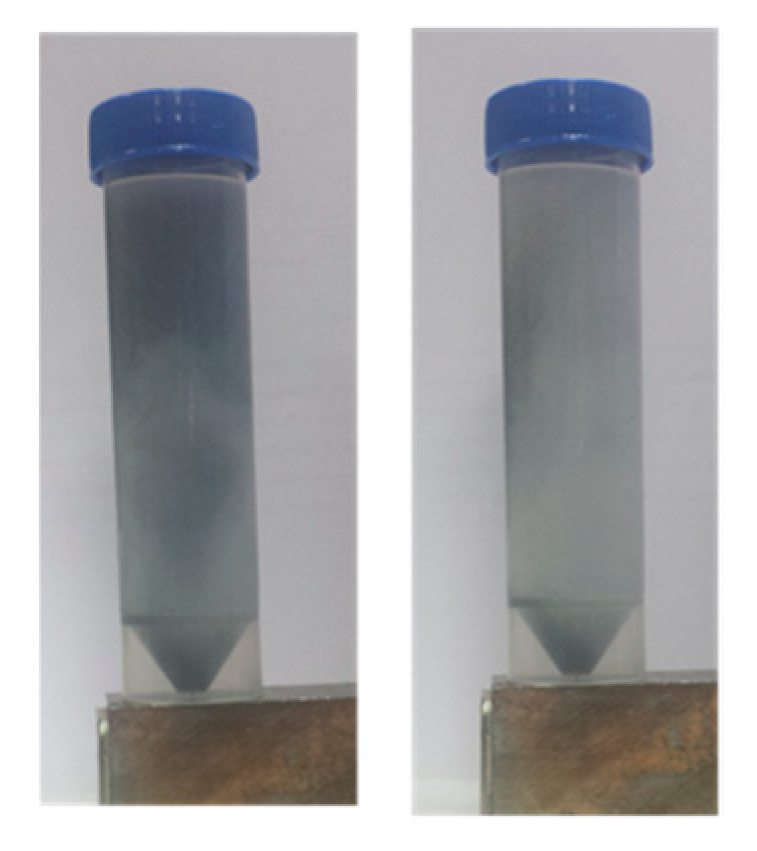
Magnetic separation of nanocomposites.

AgCl nanoparticles were separated and re-used in this work, but the process took several stages to complete. Thus, a preliminary study was carried out to analyze the efficiency of magnetic nanocomposites, since the industrial recovery of the catalyst would be facilitated. Thus, Fe_3_O_4_ was combined with AgCl and also TiO_2_ due to its great degradation capacity associated with a difficult separation [65,66]. For comparative purposes, the same degradation conditions (10 ppm ATL, 0.75 g·L^−1^ nanocatalyst) were selected. Figure 17 shows the results obtained with these nanocomposites and the corresponding nanoparticles. Some degradation results are numerically presented in Table 2. As shown in Section 3.3.1, AgCl and TiO_2_ nanoparticles offer similar degradation results, with a slightly higher velocity in the case of the latter, and degradation obtained with Fe_2_O_3_ is very limited. Regarding nanocomposites, it can be observed that they present an intermediate behavior between their two constituents. Thus, comparing nanocomposites and nanoparticles, the latter show higher degradation capacity. This was expected because at the same concentration of the nanocatalyst, nanocomposites have Fe_3_O_4_ with low photocatalytic activity. However, these nanocomposites have a great advantage in the separation process and subsequent reuse. Their magnetic properties allow for an easy separation with the help of a simple magnetic field, thus avoiding the expensive process of ultracentrifugation. This is shown in Figure 18. A practically complete separation from water was achieved in a matter of seconds using a simple magnet.

Comparing AgCl@Fe_2_O_3_ and TiO_2_@Fe_3_O_4_ nanocomposites, the first clearly led to better results. After 30 min of reaction, the degradation achieved with AgCl@Fe_3_O_4_ MNCs is 66% and with TiO_2_@Fe_3_O_4_ is 44% (see Table 2). Moreover, total degradation was not possible in this last case, whereas the nanocomposite with silver achieved it in about 95 min.

## 4. Conclusions

The use of the IL [P_6,6,6,14_]Cl allowed the preparation of simple nanoparticles such as AgCl and also more complex nanocomposites (AgCl@Fe_2_O_3_ and TiO_2_@Fe_3_O_4_) at room pressure and low temperatures without the need for any other solvent. Among many other applications, the use of these nanomaterials as photocatalysts in the degradation of emerging pollutants is promising.

Selecting ATL as a model of a pharmaceutical pollutant in wastewater, it was found that UV-C irradiation and a catalyst are required to achieve competitive results. The use of 0.75 g·L^−1^ of AgCl nanoparticles led to a degradation of ATL of 96% in aqueous solution (10 ppm) after 30 min and a practically total degradation in about 45 min. Slightly better results were obtained with TiO_2_ nanoparticles but separation was more difficult.

Selecting AgCl as a nanocatalyst, it was found that the increase in its concentration accelerates the degradation process. Higher concentrations of ATL in the wastewater mean lower degradation if the nanocatalyst concentration is fixed. A natural pH of the solution (5.5) led to the greatest degradation rates. The use of oxidizing agents slightly increases the degradation rate, at the expense of increasing the risk and cost of the process. The catalyst can be separated by centrifugation and re-used at least three times with a small loss of efficiency. The degradation process follows first-order kinetics. The main reactions occurring during degradation were ipso-hydroxylation and subsequent fragmentation, hydroxylation with detachment of the amide moiety and further oxidation, and introduction of hydroxyl groups into the aromatic ring or the alkyl chain. 

Magnetic composites (AgCl@Fe_2_O_3_ and TiO_2_@Fe_3_O_4_) led to worse degradation results than AgCl or TiO_2_ due to the limited photocatalytic activity of Fe_3_O_4_. Of the two, the silver nanocomposites clearly performed better and total degradation was achieved after about 95 min, but this was far slower than in the case of AgCl alone. However, the nanocomposites have a great advantage in the separation process, since their magnetism makes separation simple and quick.

## Figures and Tables

**Table 1:** List of ATL degradation compounds and their identification parameters.

Product	Experimental m/z	Proposed Formula	Mass Error (ppm)	Score
Atenolol	267.1705	C_14_H_22_O_3_N_2_	−0.33	99.92
A-1	283.1648	C_14_H_22_O_4_N_2_	3.48	92.39
A-2	299.1595	C_14_H_22_O_5_N_2_	2.05	85.5
A-3	281.1499	C_14_H_20_O_4_N_2_	−1	86.34
A-4	152.0703	C_8_H_9_O_2_N	0.97	85.13
A-5	134.1178	C_6_H_15_O_2_N	−1.46	87.74
A-6	238.1439	C_13_H_19_O_3_N	0.96	95.32
A-7	254.1387	C_13_H_19_O_4_N	3.02	89.79

**Table 2:** Degradation achieved by different nanocatalysts after 30 min under UV–Vis light (Initial concentration of ATL, 10 ppm; nanomaterials, 0.75 g/L).

Catalyst	Size (nm)	Degradation—30 min (%)
AgCl	<20	95.7
TiO_2_	<21	96.1
Fe_3_O_4_	<100	23.4
Fe_3_O_4@_AgCl	<100	66.0
Fe_3_O_4@_TiO_2_	<100	43.7

## Supplementary Materials

The following are available online at https://www.mdpi.com/2079-4991/11/2/411/s1, Figure S1: 1H and 13C spectra of [P_6, 6, 6, 14_]Cl, Figure S2: UV-Vis-absorbance of AgCl nanoparticles, Figure S3: XRD diffraction pattern of AgCl nanoparticles, Figure S4: XPS spectra of AgCl nanoparticles: Ag3d (a) and Cl2p (b), Figure S5: EDS spectrum of TiO_2_@Fe_3_O_4_ nanocomposite, Figure S6: XRD patterns of TiO_2_@Fe_3_O_4_ nanocomposite, Figure S7: XPS spectra of TiO_2_@Fe_3_O_4_ nanocomposite: Fe2p (a) and Ti2p (b), Figure S8: EDS spectrum of AgCl@Fe_3_O_4_ nanocomposite, Figure S9: XRD patterns of AgCl@Fe_3_O_4_ nanocomposite, Figure S10: XPS spectra of AgCl@Fe_3_O_4_ nanocomposite: Ag3d (a) and Fe2p (b)
