# Supplementary material for: Nanomaterial Synthesis in Ionic Liquids and Their Use on the Photocatalytic Degradation of Emerging Pollutants
Source: Nanomaterials (Basel). 2021 Feb 5;11(2):411. doi: 10.3390/nano11020411 (PMC7916080; doi:10.3390/nano11020411)
Supplement: Supplementary file 1 [file nanomaterials-11-00411-s001.pdf]

Supplementary Material

Nanomaterial synthesis in ionic liquids and their use on the photocatalytic degradation of emerging pollutants

Raquel Corchero[1], Rosario Rodil[2], Ana Soto[1], and Eva Rodil[1*]

[1] CRETUS Institute, Department of Chemical Engineering, Universidade de Santiago, E-15782 Santiago de Compostela, Spain; raquel.corchero@rai.usc.es (RC); ana.soto@usc.es (AS)

[2] Department of Analytical Chemistry, Nutrition and Food Science, Universidade de Santiago de Compostela, E-15782 Santiago de Compostela, Spain; rosario.rodil@usc.es (RR)

* Correspondence: eva.rodil@usc.es (ER)

Index:

- **Figure S1:** ^{1}H and ^{13}C spectra of $[P_{6,6,6,14}]Cl$ S2
- **Figure S2:** UV-Vis-absorbance of AgCl nanoparticles S3
- **Figure S3:** XRD diffraction pattern of AgCl nanoparticles S3
- **Figure S4:** XPS spectra of AgCl nanoparticles: Ag3d (a) and Cl2p (b) S3
- **Figure S5:** EDS spectrum of $TiO_2@Fe_3O_4$ nanocomposite S4
- **Figure S6:** XRD patterns of $TiO_2@Fe_3O_4$ nanocomposite S4
- **Figure S7:** XPS spectra of $TiO_2@Fe_3O_4$ nanocomposite: Fe2p (a) and Ti2p (b) S4
- **Figure S8:** EDS spectrum of $AgCl@Fe_3O_4$ nanocomposite S5
- **Figure S9:** XRD patterns of $AgCl@Fe_3O_4$ nanocomposite S5
- **Figure S10:** XPS spectra of $AgCl@Fe_3O_4$ nanocomposite: Ag3d (a) and Fe2p (b) S5

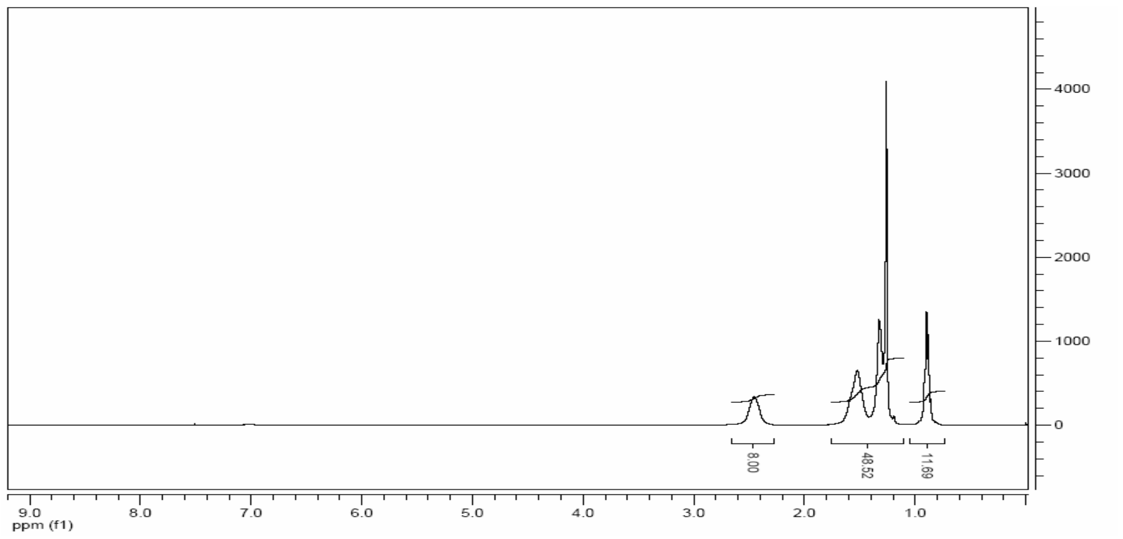

^{1}H NMR (300 MHz, $CDCl_3$) δ ppm 0.80-0.97 (unresolved, 12H, $4 \times C\underline{H}_3$), 1.15-1.70 (unresolved, 48H, $PCH_2(C\underline{H}_2)_{12}CH_3$ and $3 \times PCH_2(C\underline{H}_2)_4CH_3$), 2.35-2.65 (unresolved, 8H, $4 \times PC\underline{H}_2$).

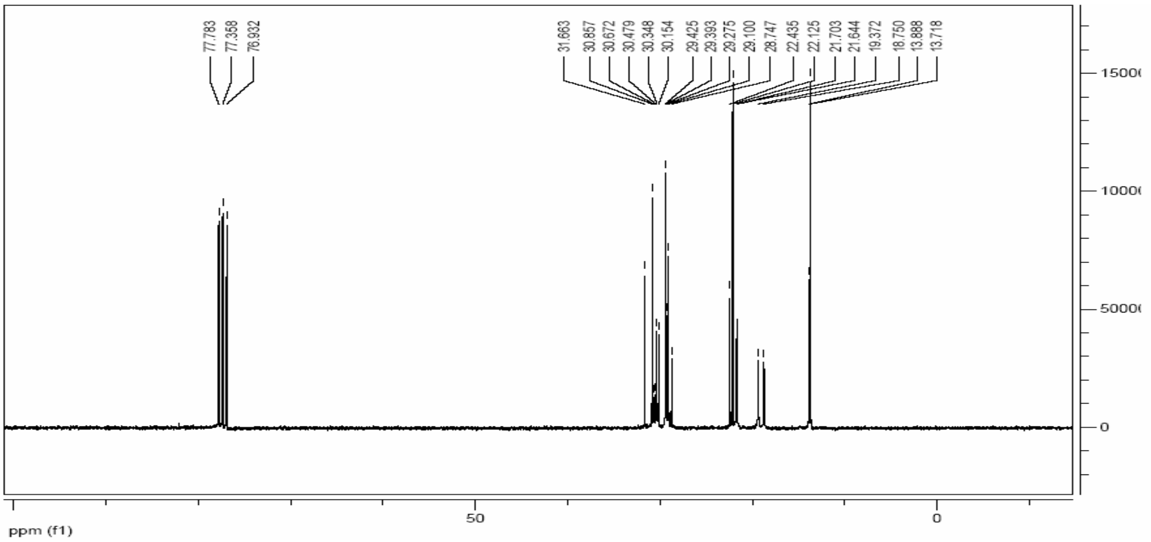

^{13}C NMR (75.4 MHz, $CDCl_3$) δ ppm 12-32 (unresolved, $P(\underline{C}H_2)_{13}\underline{C}H_3$ and $3 \times P(\underline{C}H_2)_5\underline{C}H_3$).

Figure S1: ^{1}H and ^{13}C spectra of $[P_{6,6,6,14}]Cl$

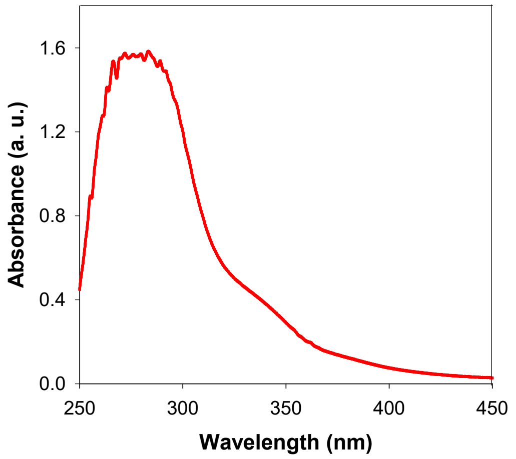

Figure S2: UV-Vis-absorbance of AgCl nanoparticles

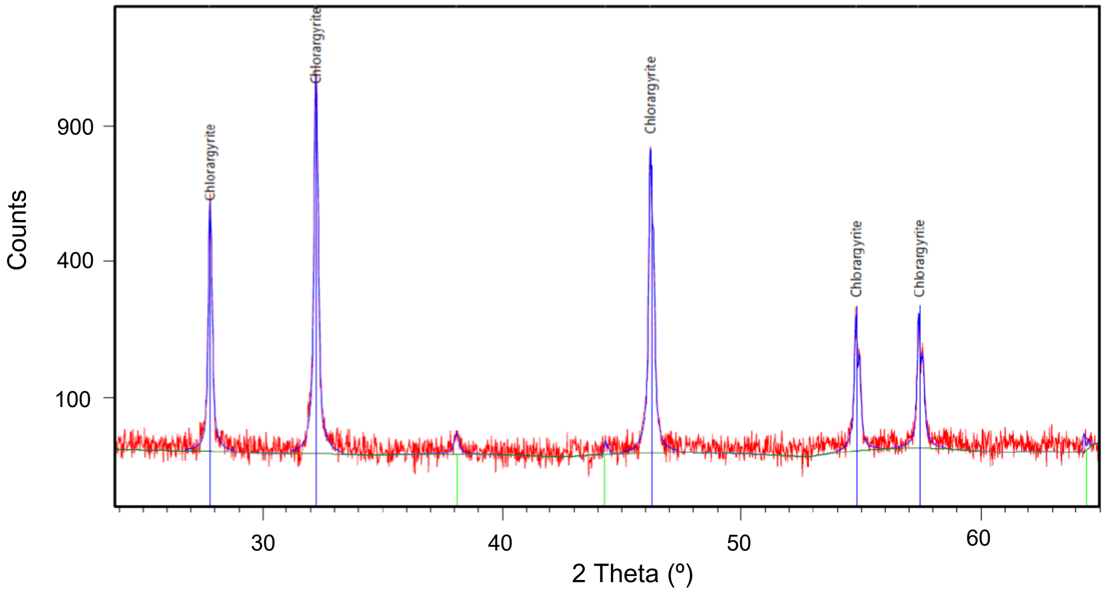

Figure S3: XRD diffraction pattern of AgCl nanoparticles

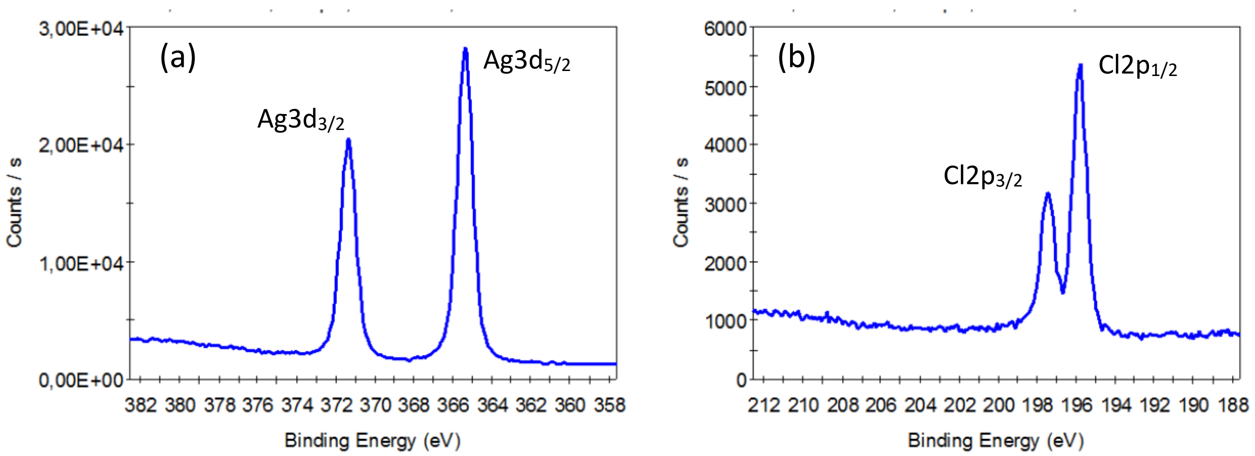

Figure S4: XPS spectra of AgCl nanoparticles: Ag3d (a) and Cl2p (b) scans

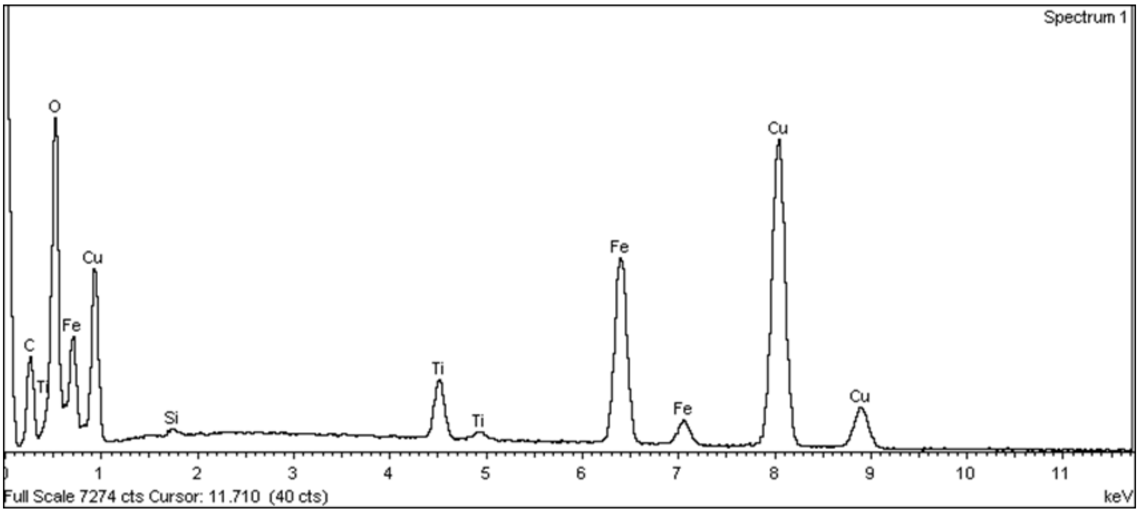

Figure S5: EDS spectrum of $TiO_2@Fe_3O_4$ nanocomposite

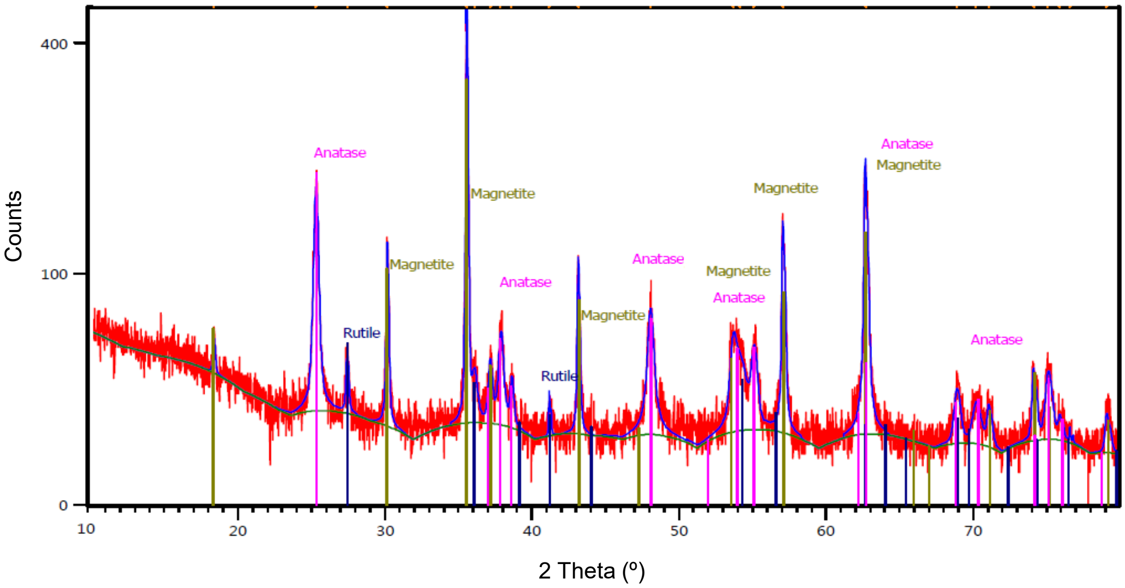

Figure S6: XRD patterns of $TiO_2@Fe_3O_4$ nanocomposite

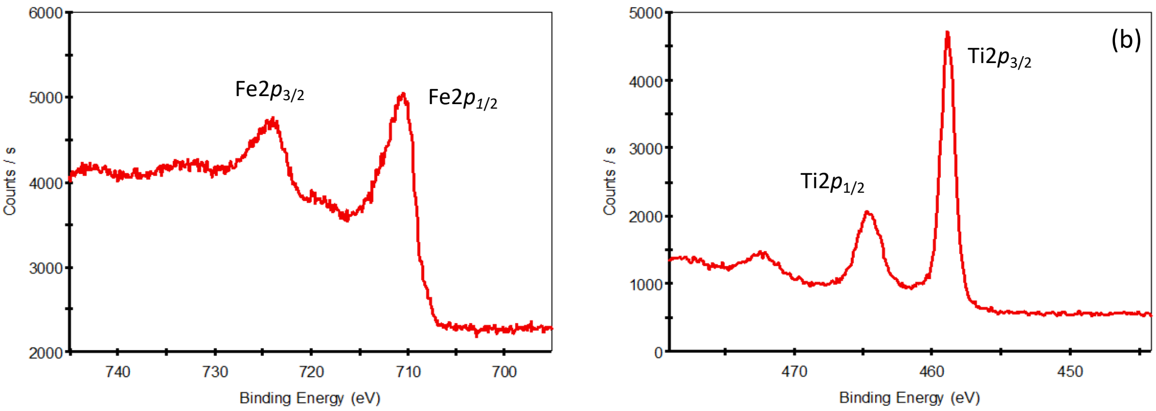

Figure S7: XPS spectra of $TiO_2@Fe_3O_4$ nanocomposite: Fe2p (a) and Ti2p (b)

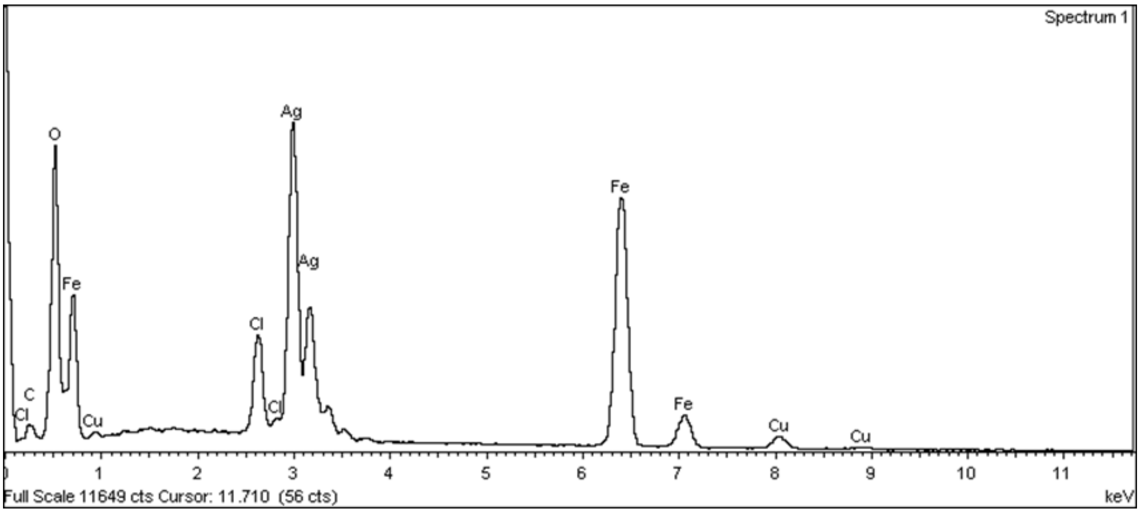

Figure S8: EDS spectrum of $AgCl@Fe_3O_4$ nanocomposite

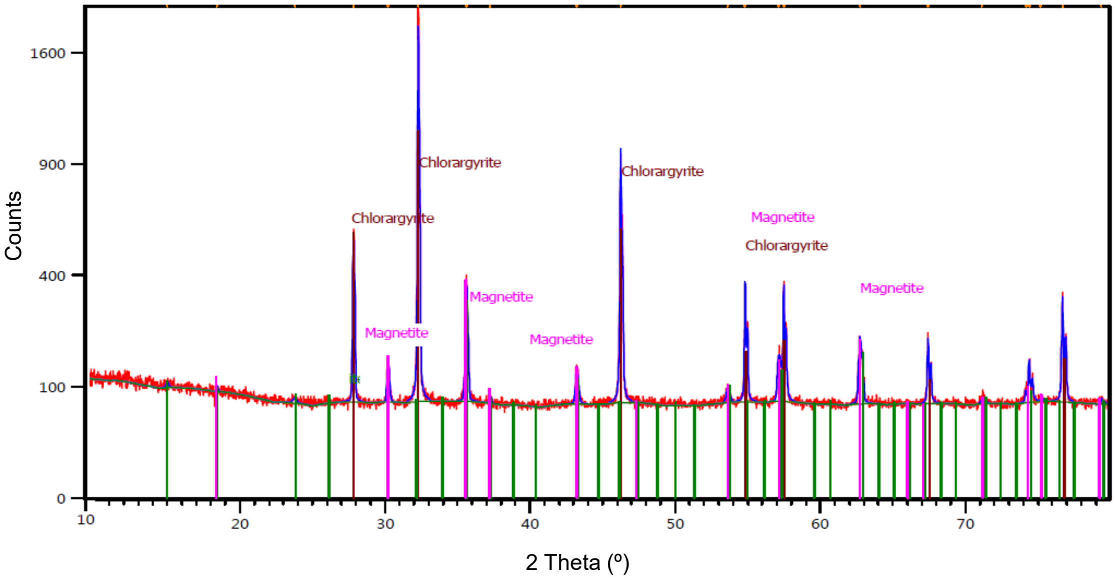

Figure S9: XRD patterns of $AgCl@Fe_3O_4$ nanocomposite

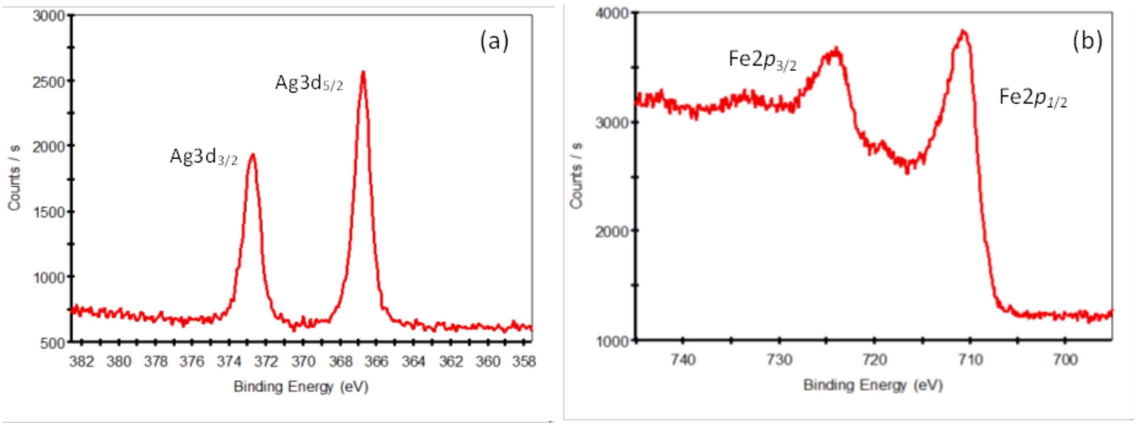

Figure S10: XPS spectra of $AgCl@Fe_3O_4$ nanocomposite: Ag3d (a) and Fe2p (b)
